# *SIK2* promotes malignant features of human osteosarcoma via up-regulating MMP2 and β-catenin expression

**DOI:** 10.1016/j.gendis.2024.101325

**Published:** 2024-06-26

**Authors:** Yutong Wang, Yulan Yao, Sen Kou, Shanshan Wang, Juntao Song, Siqi Yang, Hongwei Wang, Yunliang Wang

**Affiliations:** a School of Public Health and Laboratory of Qilu Medical University, Zibo, Shandong 255300, China; b Department of Neurology, The Second Affiliated Hospital of Zhengzhou University, Zhengzhou, Henan 450014, China; c Department of Neurology, Zibo 148 Hospital, Zibo, Shandong 255300, China; d Department of Oncology and Hematology, Zibo 148 Hospital, Zibo, Shandong 255300, China; e School of Pharmacy, Nanjing Medical University, Nanjing, Jiangsu 211166, China

Osteosarcoma is a primary malignant bone tumor that commonly occurs in children and adolescents. It is highly malignant and prone to metastasis, with a very poor prognosis. There is currently no effective treatment for this disease.^1^

*SIK2* (salt-inducible kinase 2), which is located on human chromosome 11, belongs to the AMPK (AMP-activated protein kinase) family that regulates energy metabolism.^2^ Previous studies have found that *SIK2* protein participates in a variety of intracellular signaling pathways by interacting with a variety of intracellular functional proteins, including those involved in tumorigenesis, metastasis, and apoptosis. *SIK2* has been shown to act as a potential tumor promoter in ovarian, prostate, and colorectal cancers. In contrast, *SIK2* may serve as a tumor suppressor in gastric cancer and pancreatic ductal adenocarcinoma.^3^ However, irrespective of this dichotomy, the function and mechanism of *SIK2* in osteosarcoma tumorigenesis and progression have yet to be elucidated. In this study, we constructed a lentiviral vector to induce *SIK2* RNA interference (RNAi) and observed its effects on the biological behavior of human osteosarcoma cells. The mechanism by which *SIK2* inhibits the proliferation, migration, and invasion of osteosarcoma was also explored for the first time.

We based the Kaplan-Meier survival analysis performed on a dataset of 42 samples in the GEO database. The results showed significant differences in overall survival associated with *SIK2* gene expression. A significant separation in survival curves was observed, indicating a distinct survival benefit for individuals with lower *SIK2* expression (*P* = 0.0419). Notably, the survival probability for individuals with lower *SIK2* expression remained consistently higher over the observed period, emphasizing the gene's potential prognostic relevance. The number of at-risk individuals in each group, denoted as "<16.47" (*n* = 21) and ">17.28" (*n* = 21), highlights the balanced distribution of sample sizes. Notably, individuals with higher *SIK2* expression demonstrated a decreased survival probability over the observed period, contrasting sharply with the better survival outcomes in the low expression group (Fig. 1A).

**Figure 1:**
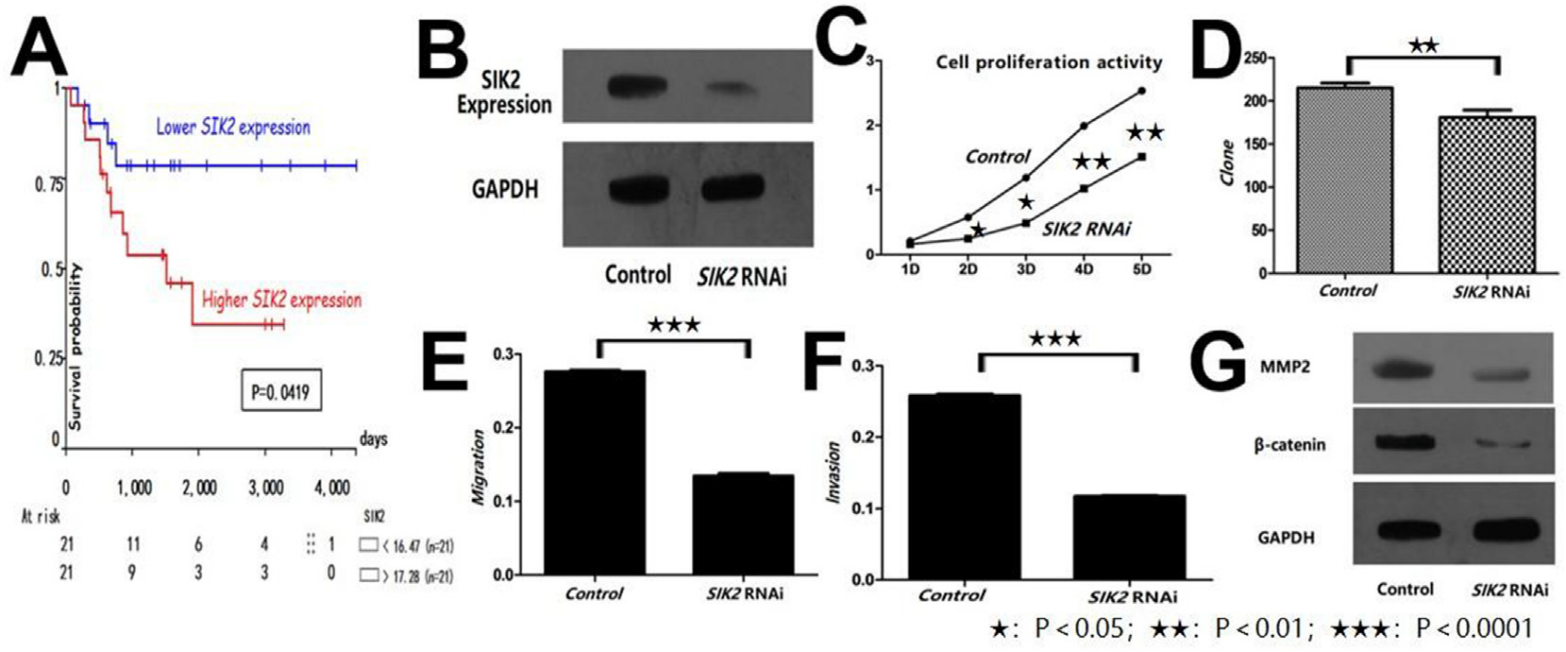
The effects of *SIK2* on human osteosarcoma U2OS cells. **(A)** Kaplan-Meier survival analysis of 42 samples based on *SIK2* gene expression. **(B)** Protein expression of *SIK2* and *GAPDH* (reference gene) in the two groups. **(C)** Cell proliferation activity (assayed by cell counting kit-8) at 2–5 days (all *P* < 0.05). **(D)** Number of clonal cells in the two groups (*P* = 0.0069). **(E)** Number of migrating cells in the two groups (*P* < 0.0001). **(F)** Number of invasive cells in the two groups (*P* < 0.0001). **(G)** Protein expression of MMP2 and β-catenin in the two groups. SIK2, salt-inducible kinase 2; GAPDH, glyceraldehyde-3-phosphate dehydrogenase; MMP2, matrix metalloproteinase 2; RNAi, RNA interference.

We digested and ligated the shRNA and pGCSIL-GFP, prepared and identified clones, and then used 293T cells to package the virus, and the titer was determined by a hole-by-hole dilution method. On the fifth day after the virus was transfected into U2OS cells (human bone osteosarcoma epithelial cells), more than 90% of the cells expressed green fluorescent protein under a fluorescence microscope. The target cells infected with the lentivirus *SIK2* RNAi negative control served as the control group, and the target cells infected with lentivirus *SIK2* RNAi were the experimental group (*SIK2* RNAi group). Cell culture and virus transfection were carried out for 72 h after transfection, then real-time quantitative PCR was used to detect *SIK2* and *GAPDH* (glyceraldehyde-3-phosphate dehydrogenase) expression and the relative mRNA expression of *SIK2* was determined. The results showed that the amplification system was good, the peak value of the melting curve was high and sharp, there were no stray bands, and the amplification product was specific. The relative *SIK2* mRNA expression in two groups was calculated by the 2^−ΔΔCt^ method. The expression of SIK2 mRNA in the *SIK2* RNAi group was significantly lower than that in the control group (*P* = 0.0227). The knockdown efficiency in the *SIK2* RNAi group was more than 70%, which indicated that the recombinant lentivirus could specifically inhibit the expression of *SIK2*. Western blotting was used to detect the protein expression of *SIK2* and *GAPDH* in the *SIK2* RNAi group and control group, and a gel imaging system was used to scan the films. The results showed that the expression of *SIK2* protein in the *SIK2* RNAi group was significantly lower than that in the control group (Fig. 1B).

We analyzed the cell proliferation activity by the cell counting kit-8 method. The results showed that when U2OS cells grew for 2–5 days, the absorbance of the *SIK2* RNAi group was significantly lower than that of the control group (All *P* < 0.05) (Fig. 1C). Thus, siRNA-mediated *SIK2* knockdown can inhibit the proliferation of U2OS cells.

We cloned U2OS cells from the control and experimental groups. After the cells were infected with lentivirus, the cell clone formation was detected using a Cellomics kit. The number of cells was 196.3333 ± 4.93 in the *SIK2* RNAi group and 215.3333 ± 3.18 in the control group (Fig. 1D). Thus, the clone formation in the *SIK2* RNAi group was significantly decreased compared with the control group.

We then used the transwell assay to detect the migration of U2OS cells. The results showed that the 570-nm absorbance of U2OS cells in the *SIK2* RNAi group was 0.2760 ± 0.0021, which was significantly lower than that in the control group (0.1345 ± 0.0036) (*P* < 0.0001; Fig. 1E). These results suggested that the siRNA-mediated reduction in the expression of *SIK2* gene could effectively inhibit the migration of U2OS cells.

We also used the transwell assay to detect the invasion of U2OS cells. The results showed that the 570-nm absorbance of U2OS cells in the control group and *SIK2* RNAi group was 0.2760 ± 0.0021 and 0.1345 ± 0.0036, respectively, which was significantly different (*P* < 0.0001) (Fig. 1F). Therefore, using siRNA to inhibit the expression of *SIK2* could effectively reduce the invasion of U2OS cells.

β-catenin is an important multifunctional cytoskeletal protein encoded by the gene *CTNNB1* (catenin beta 1). It not only mediates intercellular adhesion and signal transduction but also plays a central role in regulating the Wnt signaling pathway. It can regulate many target genes related to tumor cell proliferation, invasion, and metastasis by interacting with diverse transcriptional activators.^4^ MMP2 (matrix metalloproteinase 2) is a zinc-dependent proteolytic enzyme. By acting on type IV and V collagens, important components of the extracellular matrix, MMP2 destroys the histological barrier of tumor cell invasion and promotes tumor cell invasion and metastasis.^5^

We next examined the protein expression in U2OS cells from each group by western blotting. The results showed that the expression levels of MMP-2 and β-catenin in U2OS cells transfected with *SIK2* RNAi were significantly lower than those in the control (Fig. 1G). This suggests that inhibiting the expression of *SIK2* can effectively inhibit the migration and invasion of human osteosarcoma cells.

The purpose of this study was to explore the effects of *SIK2* on the malignant activity of osteosarcoma cells. The cell counting kit-8 and colony formation assays showed that the proliferation and clone formation of the *SIK2* RNAi group were significantly lower than those of the control group. The transwell assay showed that the ability of cells to migrate and invade in the *SIK2* RNAi group was significantly weaker than that in the control group. Together, the results show that RNA interference can significantly inhibit the proliferation and invasion of U2OS cells, and *SIK2* may thus play an important role in the occurrence and development of osteosarcoma.

To understand the possible mechanism, the expression of related proteins in U2OS cells was detected by western blotting after RNA interference inhibited the expression of *SIK2*. The results showed that *SIK2* gene silencing down-regulated the expression of MMP-2 and β-catenin, suggesting that these factors may work together to play an important role in the occurrence, development, invasion, and metastasis of osteosarcoma.

In this study, lentivirus-mediated RNA interference specifically inhibited the expression of *SIK2* and significantly inhibited the proliferation and invasion of osteosarcoma cells, suggesting that *SIK2* plays an important role in the occurrence, development, invasion, and metastasis of osteosarcoma. *SIK2* may be a potential molecular target for osteosarcoma therapy, and the present findings provide new information about the development and progression of osteosarcoma.

## Author contributions

Yutong Wang and Yulan Yao carried out the experiments, collected the data, and drafted the manuscript. Sen Kou, Shanshan Wang, Juntao Song, and Siqi Yang provided resources and performed the data analysis. Yunliang Wang and Hongwei Wang revised/edited the manuscript. All authors read and approved the submitted manuscript.

## Conflict of interests

The authors declared no conflict of interests.
