# Supplementary material for: SIK2 promotes malignant features of human osteosarcoma via up-regulating MMP2 and β-catenin expression
Source: Genes Dis. 2024 Jun 26;12(2):101325. doi: 10.1016/j.gendis.2024.101325 (PMC11616025; doi:10.1016/j.gendis.2024.101325)

**Editing Certificate**

**
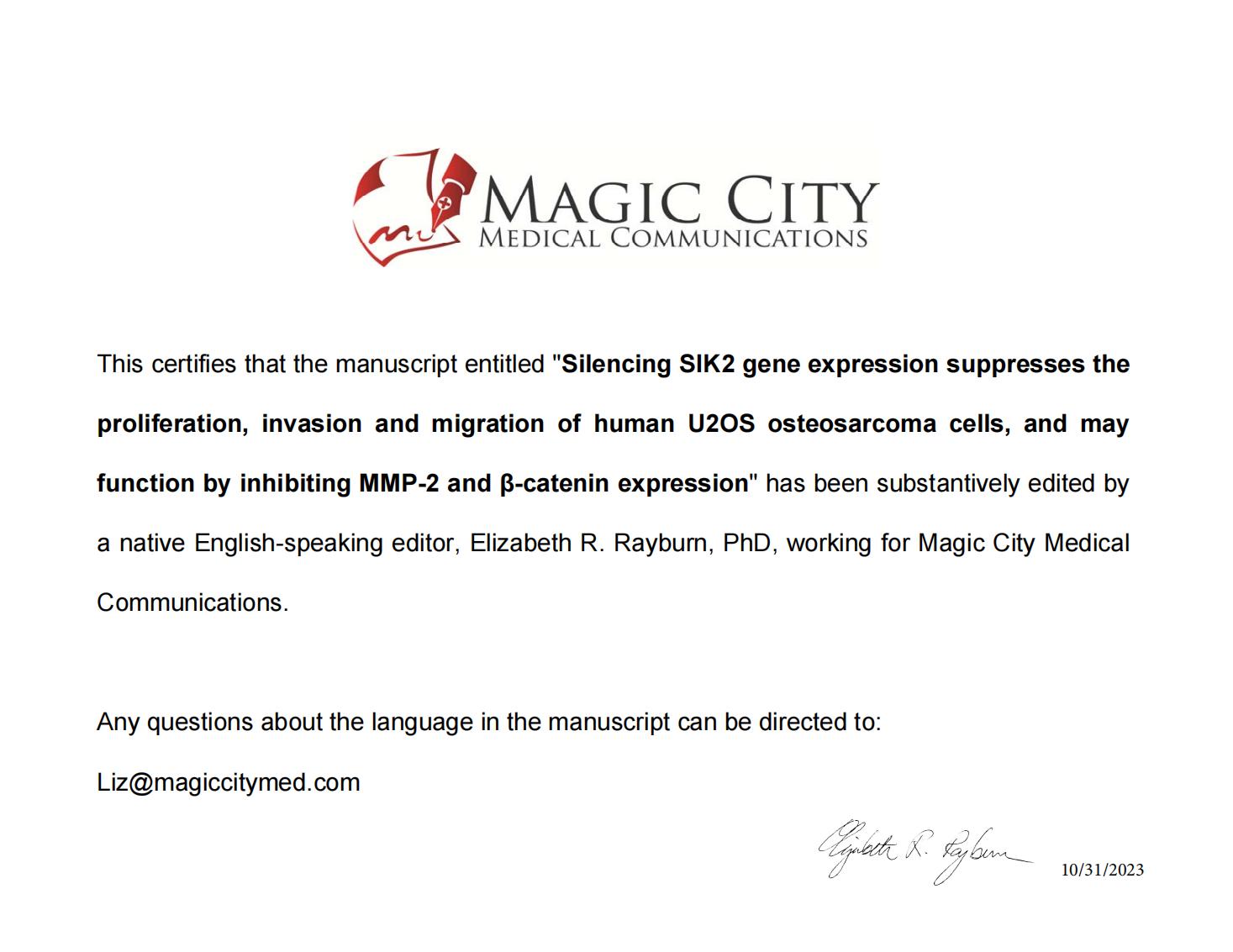
**

**Meterials**

1. Main experimental reagents

The human osteosarcoma U2OS cell line was purchased from the Cell Resource Center of Shanghai Institutes for Biological Sciences, Chinese Academy of Sciences. Renal epithelial 293T cells and E. coli strain DH5α, interference sequences, primers, pGCSIL-GFP and other plasmids were purchased from Shanghai Ruisai Biotechnology Co., Ltd; Transfection reagents Lipofectamine 2000 and TrizolRNA extraction kits were purchased from Invitrogen Company of the United States; CCK-8 is purchased from Tongren Institute of Chemistry in Japan, and trypsin is purchased from Shanghai Chemical Reagent Company; M-MLv reverse transcriptase, dNTP and RNasin were purchased from promega Company of USA; Mouse anti-human MMP-2 and β-catenin antibodies purchased from Abcam Company; Matrigel matrix glue is purchased from BD Company of USA. The rest of the reagents are imported or domestic.

**Methods**

1. Immunohistochemical detection of SIK2 protein expression

The paraffin-embedded tissue specimens were sliced, dewaxed and put into water according to the thickness of 4 μ m. The slices were immersed in 3%H2O2 solution and incubated at room temperature for 10 minutes to block the activity of endogenous peroxidase. The slices were immersed in 0.01MPH=6.0 citrate solution and heated in 500W microwave oven for 10 minutes. 5% normal goat serum was added to the section and sealed at room temperature for 20 minutes to reduce the probability of non-specific staining. Get rid of the excess liquid, properly add mouse anti-human SIK2 polyclonal antibody (working concentration 1: 200) and incubate at 37 ℃ for 1 h. Wash 2min with PBS, repeat for 3 times, and incubate 30min with second anti-goat anti-human IgG,37 ℃. DAB (3jin3-diaminobenzidine tetrachloride) was stained with hematoxylin, the positive expression was brown and granular, the positive expression of SIK2 protein was observed under the mirror under the microscope, each section was observed under the high power lens field (10 × 40) and 5 non-repetitive visual fields were randomly selected, and the average optical density of staining under each visual field was measured by image analysis software.

2. SIK2 gene RNA interferes with the preparation of lentiviral vector and infects cells

Lentiviral vector plasmids PGCSIL-GFP, pHelp1.0 and pHelp2.0 were purchased from Shanghai Ruisai Genochemistry Co., Ltd. The siRNA sequence is designed by commercial software (Applied Biosystems/Ambion,TX, USA). For SIK2,siRNA, the sense sequence is listed as 5-CUACCGAGAAGUACAAAUADTDT-3', antisense sequence as 5 words UAUGUACUUCGGUAGdTdTmur3'. The siRNA sequences used as negative control were 5'- GCCUCAGCUGCGCGCGCCdTdT-3' (meaningful) and 5 'murmurGCGUCGCAGCUGGGCCAdTdTmur3' (antisense). The selected sequences were confirmed by NCBI BLAST (http://www.ncbi.nlm.nih.gov/blast/) to ensure that the selected genes were specifically targeted. S hRNA and pGCSIL-GFP were digested and ligated, and the clones were prepared and identified. Three kinds of DNA in lentivirus packaging system were extracted with plasmid extraction kit of Qiagen company. The virus was packaged with 293T cells, and the titer was determined by hole-by-hole dilution method. Five days later, the infection efficiency was determined by observing and taking pictures under the fluorescence microscope.

3. Cell culture and virus transfection

RNAi experimental group: Human osteosarcoma U2OS cell line was cultured in RPMI1640 medium containing 10%FBS and digested with trypsin in logarithmic growth phase. Human osteosarcoma U2OS cells were inoculated in a 6-well plate with 1 × 10 ~ 5 cells per well and cultured for 24 hours. when the cell confluence reached 50% of the cell growth, the lentivirus carrying SIK2-siRNA and scr-siRNA (irrelevant sequence) were transfected into U2OS cells according to Lipofectamine2000 instructions. 72 hours after transfection, green fluorescence was observed under inverted fluorescence microscope.

4. Analysis of cell proliferation activity by CCK- 8 method

The cell suspension after virus transfection was added to 96-well plank, and the inoculation density was about 3000 / well, with 3 multiple holes in each group, and cultured in 37 ℃, saturated humidity and 5%CO2 incubator. 24 h, 48 h, 72h72h, 96h96h and 120 h after the seed plate, 10 μ 1 CCK-8 reagent was added and incubated in the incubator for 3 hours. The absorbance values of each hole at different time were measured at the 450nm wavelength of the enzyme labeling instrument, and the cell proliferation curve was drawn.

5. Cell clone formation experiment

Human osteosarcoma fine U2OS cells were taken from each group in logarithmic growth phase. After digestion, re-suspension and counting, the cells were inoculated into a 6-well plate at the concentration of 1000 cells / well, gently shaken so that the cells dispersed evenly, and then incubated in a cell incubator for about 5 days, and the growth of the cells was observed every day. When the clones visible to the naked eye appeared in the petri dish, the culture was terminated and the supernatant was discarded. 0.5% crystal violet staining was fixed with methanol for 15 minutes, and the number of cell clones was observed and recorded under the microscope for statistical analysis.

6. Detection of migration and invasion of U2OS cells by Transwell assay

The U2OS cells of each group were suspended in serum-free DMEM medium 24 hours after siRNA transfection, and the concentration was adjusted to 3 × 10 ~ 6 cells / ml by cell counting board. 100 μ l of cell suspension was added to the upper chamber of the Transwell chamber (Matrigel glue was applied in the invasion experiment, but not in the migration experiment), and 600 μ l DMEM medium containing 10% fetal bovine serum was added to the lower chamber, which was routinely cultured at 37 ℃ for 24 hours with 5%CO2. The 20min was fixed with methanol, and the 10min was stained with 0.5% crystal violet. The crystal violet in the adherent cells in the lower layer of the chamber was dissolved in the lysate, and 100 μ l of blue-purple clarification solution was absorbed into the 96-well plate. The absorbance at 570nm was detected by enzyme labeling instrument, and then the cell migration and invasion were calculated.

7. Western blotting detection

The transfected U2OS cells were collected and lysed in ice water bath with RIPA buffer to extract the total protein. After the protein concentration was determined by BCA method, the protein concentration was adjusted to the same in each group and separated in 10% polyacrylamide gel (50 μ g / swimming lane). The separated protein was then transferred to polyvinylidene fluoride (polyvinylidenefluoride,PVDF) membrane by wet transfer method. 5% skim milk powder sealed at room temperature for 2 hours. The membrane was incubated with horseradish peroxidase (HRP) labeled goat anti-mouse IgG second antibody (diluted by 1TBST 10000 dilution) and incubated with horseradish peroxidase (HRP)-labeled goat anti-mouse IgG second antibody (diluted by MMP-2) for 1.5 h. After incubation, the protein bands were detected by electrochemiluminescence (ECL). Electrochemiluminescence (EC) and electrochemiluminescence (ECL) were used to display protein bands and analyze the expression of MMP-2 and β-catenin protein. The second antibody labeled with horseradish peroxidase (HRP) was diluted and incubated at 37 ℃ for 1 hour. after the antibody was incubated, the protein bands were detected by electrochemiluminescence (ECL). The results were observed and analyzed.

**Supplementary Results**

1.The effects of *SIK2* on human osteosarcoma U2OS cells.


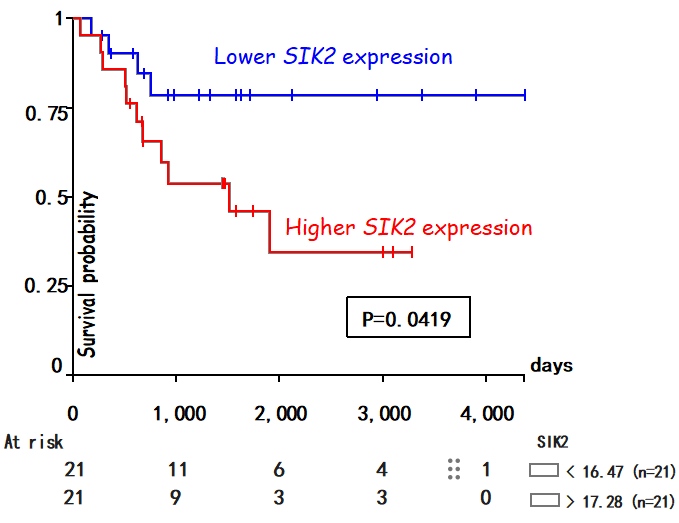


2.Principal Component Analysis of SIK2 Gene Expression in Bone Marrow Samples Across Multiple GEO Datasets.


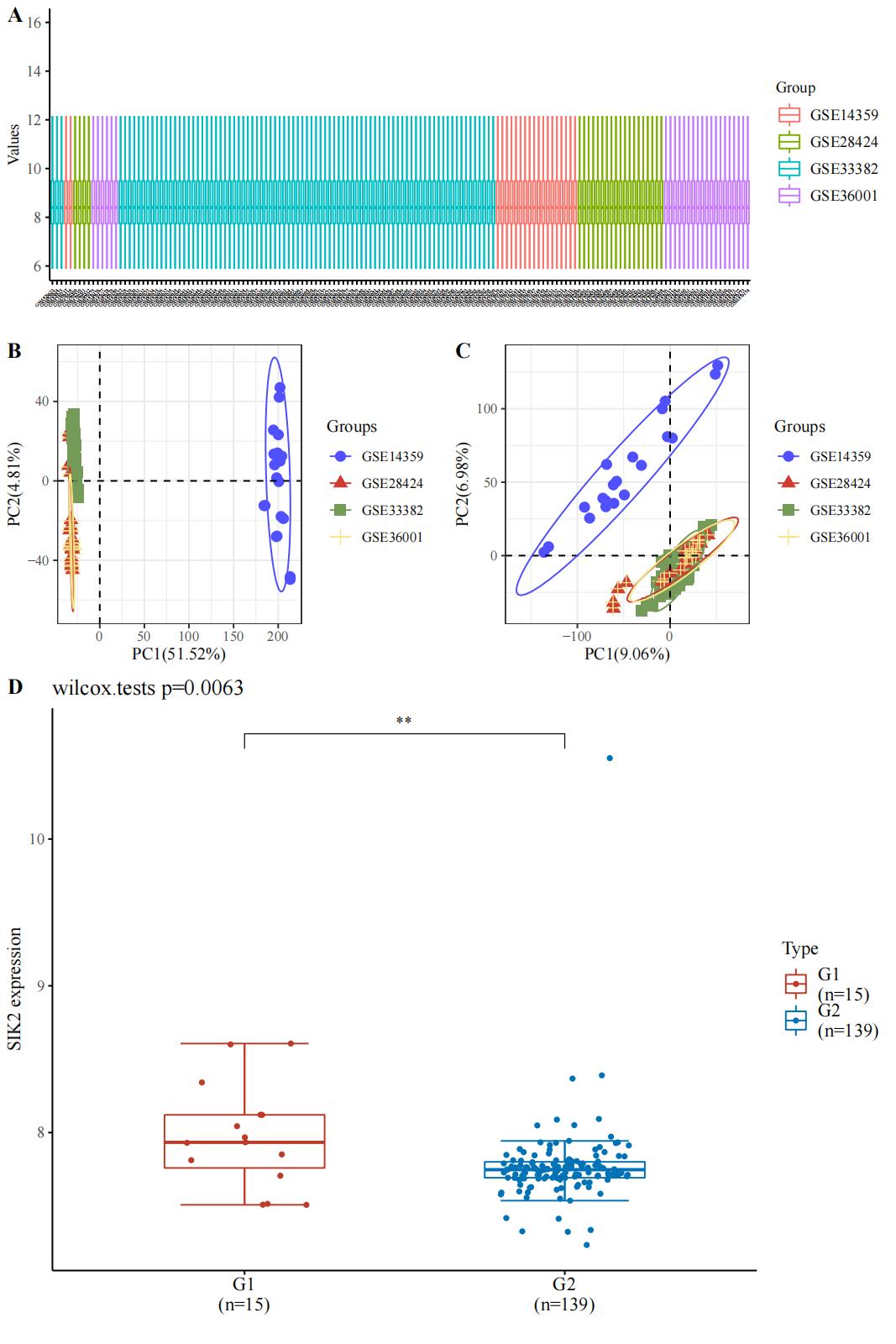


3.Single-Cell Map of Cellular Composition in a Tissue Sample Using UMAP.


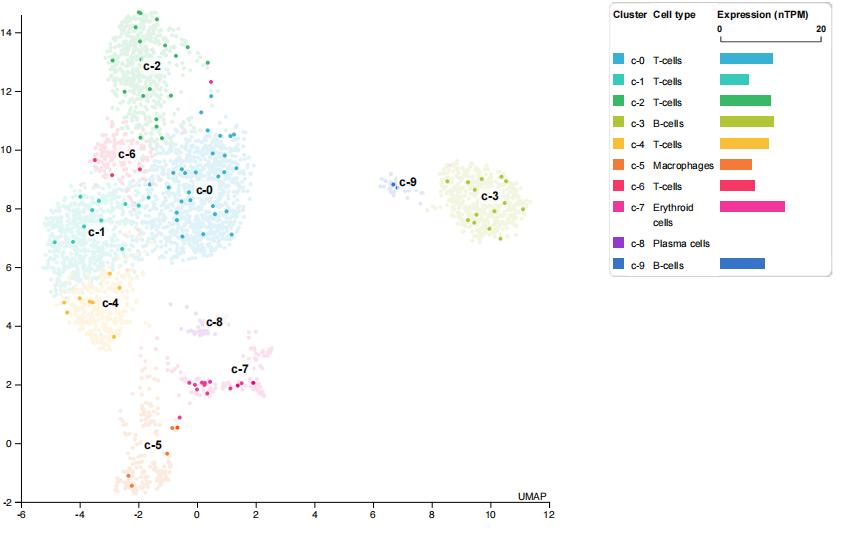


4.Single-Cell Gene Expression Analysis of SIK2 in Bone Marrow Cells.


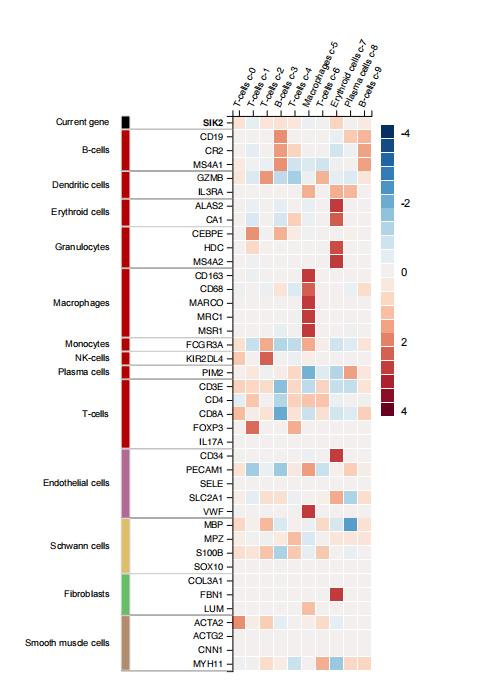


1. Immunohistochemical detection of SIK2 protein expression


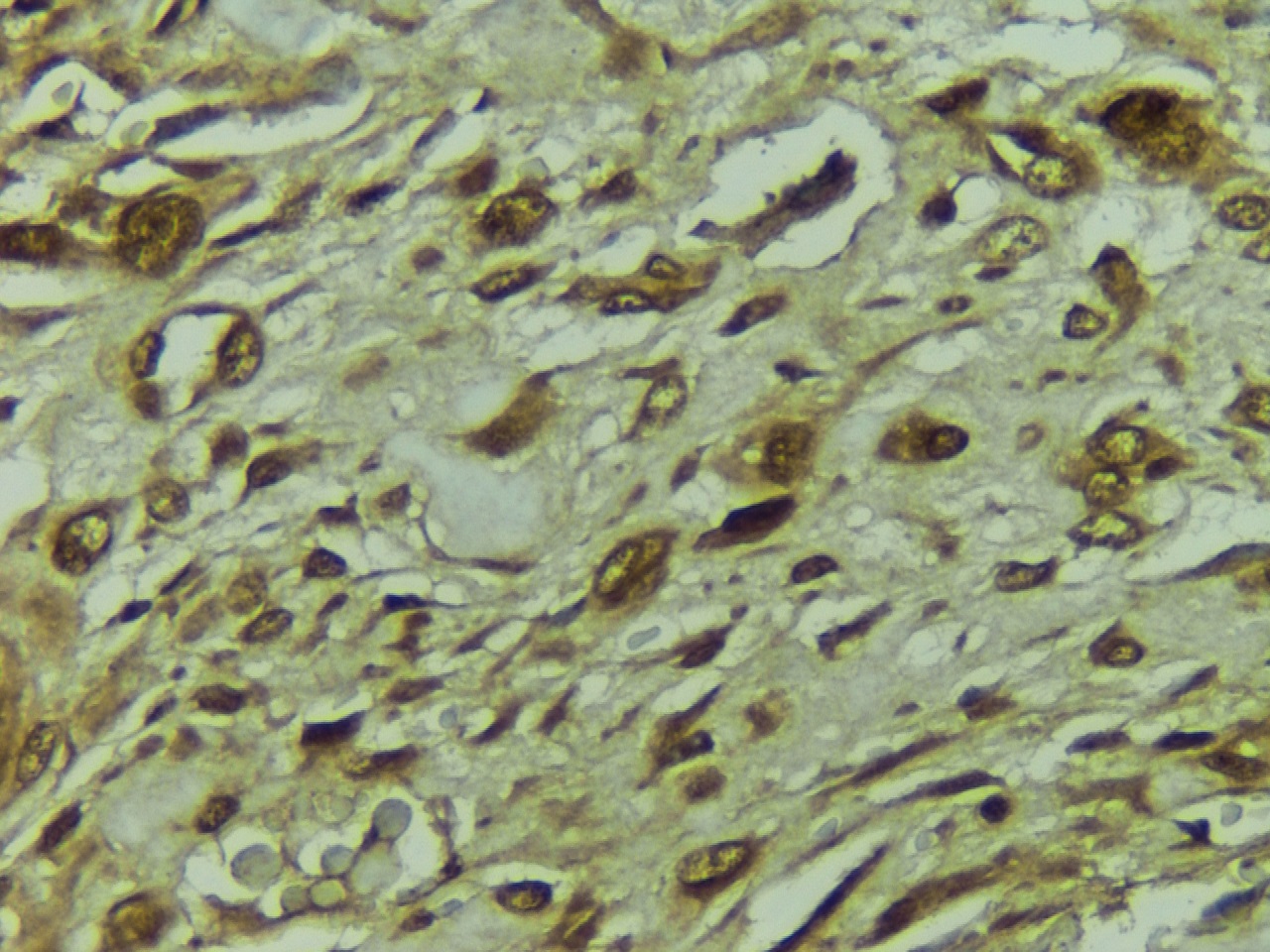


6.Protein expression of *SIK2* and GAPDH (reference gene) in the two groups.


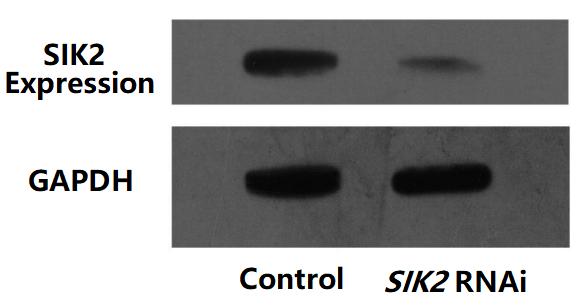


7.Cell proliferation activity by the Cell Counting Kit-8.


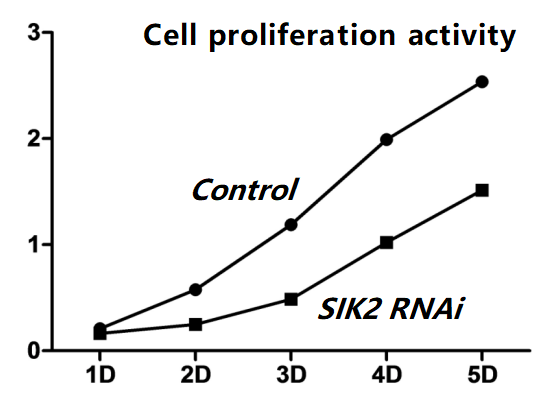


8.Number of clonal cells in the two groups (P=0.0069).


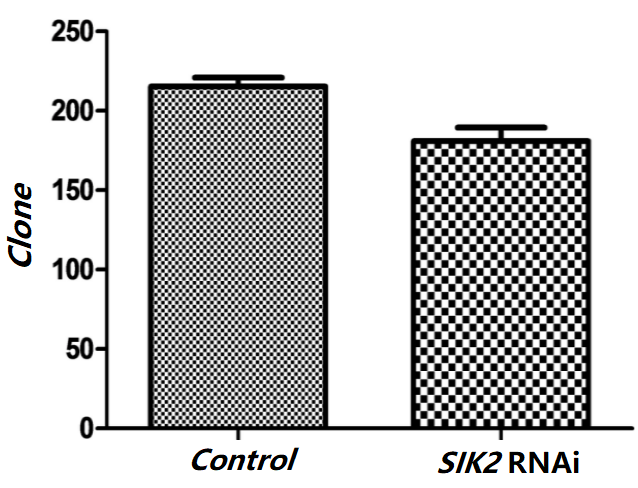

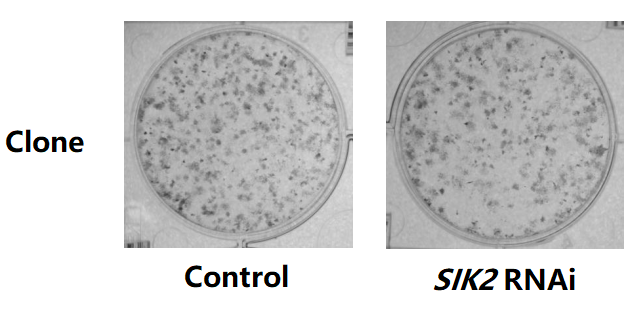


9.Number of migrating cells in the two groups (P<0.0001).


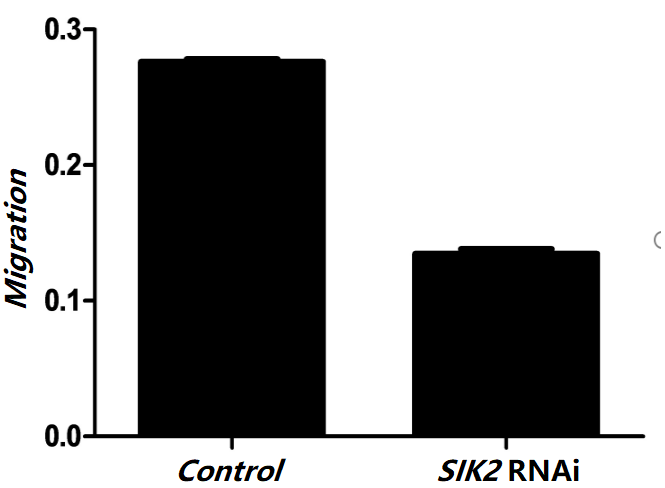

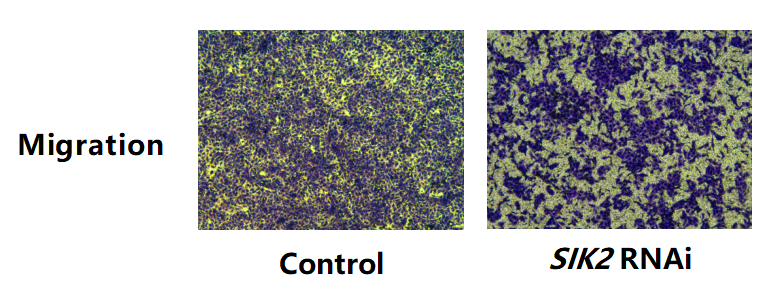


10.Number of invasive cells in the two groups (P<0.0001).


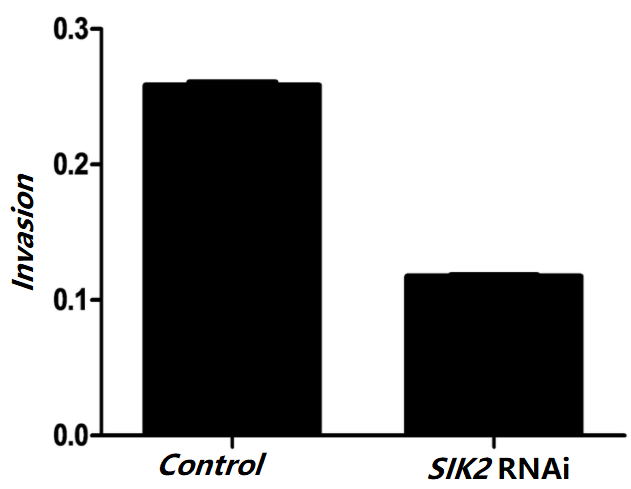

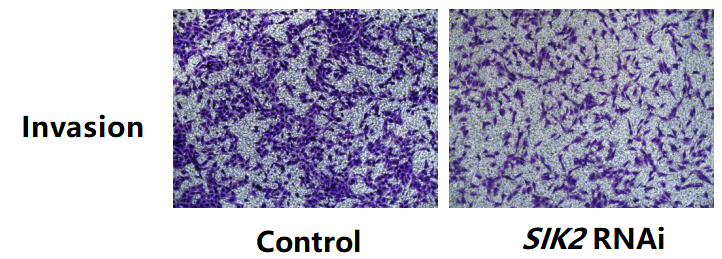


111.Protein expression of MMP2 and β-catenin in the two groups.


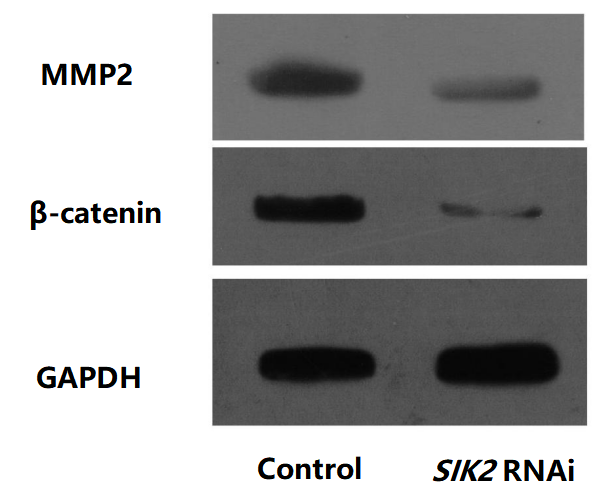

Supplement: Multimedia component 1 [file mmc1.doc]
